# Two-dimensional gel proteome reference map of human small intestine

**DOI:** 10.1186/1477-5956-7-10

**Published:** 2009-03-19

**Authors:** Maria Paola Simula, Renato Cannizzaro, Maria Dolores Marin, Alessandro Pavan, Giuseppe Toffoli, Vincenzo Canzonieri, Valli De Re

**Affiliations:** 1 Experimental and Clinical Pharmacology, CRO Centro di Riferimento Oncologico, IRCCS National Cancer Institute, via F. Gallini 2, 33081 Aviano, PN, Italy; 2 Gastroenterology, CRO Centro di Riferimento Oncologico, IRCCS National Cancer Institute, via F. Gallini 2, 33081 AVIANO, PN, Italy; 3 Pathology, CRO Centro di Riferimento Oncologico, IRCCS National Cancer Institute, via F. Gallini 2, 33081 AVIANO, PN, Italy

## Abstract

**Background:**

The small intestine is an important human organ that plays a central role in many physiological functions including digestion, absorption, secretion and defense. Duodenal pathologies include, for instance, the ulcer associated to Helicobacter Pylori infection, adenoma and, in genetically predisposed individuals, celiac disease. Alterations in the bowel reduce its capability to absorb nutrients, minerals and fat-soluble vitamins. Anemia and osteopenia or osteoporosis may develop as a consequence of vitamins malabsorption. Adenoma is a benign tumor that has the potential to become cancerous. Adult celiac disease patients present an overall risk of cancer that is almost twice than that found in the general population. These disease processes are not completely known.

To date, a two dimensional (2D) reference map of proteins expressed in human duodenal tissue is not yet available: the aim of our study was to characterize the 2D protein map, and to identify proteins of duodenal mucosa of adult individuals without duodenal illness, to create a protein database. This approach, may be useful for comparing similar protein samples in different laboratories and for the molecular characterization of intestinal pathologies without recurring to the use of surgical material.

**Results:**

The enrolled population comprised five selected samples (3 males and 2 females, aged 19 to 42), taken from 20 adult subjects, on their first visit at the gastroenterology unit for a suspected celiac disease, who did not turn to be affected by any duodenal pathology after gastrointestinal and histological evaluations. Proteins extracted from the five duodenal mucosal specimens were singly separated by 2D gel electrophoresis. After image analysis of each 2D gel, 179 protein spots, representing 145 unique proteins, from 218 spots tested, were successfully identified by MALDI-TOF ms analysis. Normalized volumes, for each protein, have been reported for every gel. Proteins have been grouped according to their biological/metabolic functions.

**Conclusion:**

This study represents to date the first detailed and reproducible 2D protein map of human duodenum. Spots identifications, reported in a database, will be helpful to identify the variability in protein expression levels, in isoforms expression, or in post-translational modifications associated to pathology or to a therapy.

## Background

The small intestine is an important human organ and plays a central role in many physiological functions including digestion, absorption, secretion and defense. The mucosa of the small intestine has various structural features, which considerably increase the luminal surface area and consequently support the main function of the small intestine: absorbing the degraded components of food. The entire intestinal mucosa arranges into two fundamental structures: the villi and the crypts of Lieberkuhun. The surface of the villi is formed predominantly by mature, absorptive enterocytes, along with occasional mucus-secreting goblet cells. Each enterocyte presents numerous microvilli on the luminal plasma membrane. The crypts of Lieberkuhun are simple tubular glands around the villi and extend through the lamina propria down to the muscularis mucosae. They are largely lined with enteroendocrine cells involved in the secretion of electrolytes, with Paneth cells releasing antibacterial substances and with stem cells, which continually divide and cause the turnover of epithelial cells. Duodenal pathology include, for instance, ulcer associated to *Helicobacter Pylori*, adenoma, cancer, and, in genetically predisposed individuals, celiac disease (CD). Alterations in the bowel diminish its capability to absorb nutrients, minerals and the fat-soluble vitamins A, D, E, and K. Anemia and osteopenia or osteoporosis may develop as a consequence of vitamins malabsorption. Adenoma is a benign tumor that has the potential to become cancerous [1]. Adult CD patients present an overall risk of cancer that is almost twice than in the general population. Cancers include T- and B cell non-Hodgkin's lymphoma and adenocarcinoma [2]. The mechanisms involved in the development of these pathologies and in tumor progression are only partially known.

Proteomics is the study of the complete protein complement of the cell or the tissue. In contrast with the genome, the proteome is dynamic and is in constant flux, due to differential splicing of the respective mRNAs, posttranslational modifications, and temporal and functional regulation of gene expression. The inherent advantage provided by proteomics is that the identified protein is itself the biological endpoint expressed into the complexity of the tissue microenvironment. Proteomics is now entering the field of biomedicine with declared expectations for the identification of new pathological markers, therapeutic targets and markers of therapeutic response. Advances in the fields of genomics and proteomics will hopefully provide insights into the molecular complexity of the disease process, thus enabling the development of tools for treatment as well as for diagnosis and prevention [3-6].

To our knowledge, a 2D gel proteome reference map with a database of human duodenum expressed proteins, has not been reported yet. The relative low frequency of duodenum routine biopsies, with respect to other anatomical sites, and the custom to fix biopsies in gastroenterological practice, have probably delayed the proteomic characterization of this tissue. Moreover proteomic approaches are not yet a routine in clinical practice. 2D maps are important prerequisites as tissue protein separation and identification represent important steps to establish the base for identifying eventual pathological perturbations [7-9]. In the present study, to contribute to the knowledge on the biology and physiology of the human small intestine, we compiled a protein expression profile of the human duodenal mucosa. We used the 2D-Difference in Gel Electrophoresis (DIGE) since this technique is sensitive and allows for less than 50 μg of protein to be successfully separated and detected, compared to the 500 μg required for a successful traditional Coomassie blue. Moreover, 2D-DIGE circumvents many of the issues associated with traditional 2D-PAGE as gel to gel variation and limited dynamic range, and allows more accurate and sensitive quantitative proteomic studies [10]. Protein spots have then been identified with matrix-assisted laser desorption/ionization-time of flight mass spectrometry (MALDI-TOF ms) and database searching. A calculation of the average protein expression levels, the standard deviation and the coefficient of variation was performed for all identified proteins. They were then grouped on the base of their biological functions.

## Methods

### Patients

For proteomic analyses duodenal biopsies were selected from five adult subjects, 3 males and 2 females, between the ages of 19 and 42, on their first visit for a suspected diagnosis of CD, attending the Gastroenterology Unit of the Centro di Riferimento Oncologico, IRCCS. Biopsies were fixed in Bouin solution and a portion of unfixed tissue was snap frozen in liquid nitrogen and stored at -80°C for proteomic analysis. Histological evaluation, according to modified Oberhuber-Marsh classification [11], excluded celiac or other pathological disorders in these specimens. HLA DQB1 PCR amplification and nucleotide sequences were carried out as previously reported [12]. Subjects were made aware of the purpose of the study and an informed consent was obtained from all the enrolled patients.

### Sample preparation for 2D-polyacrylamide gel electrophoresis

Proteins were extracted from gut biopsies with Sample grinding kit (GE Healthcare, Milan, IT) and 200 μl of lysis buffer containing 7 M urea, 2 M thiourea, 4% CHAPS and 30 mM Tris-HCl pH 8.5. The cell lysates were then prepared for 2D-DIGE with 2D Clean-Up kit (GE Healthcare) and resuspended in 7 M urea, 2 M thiourea and 4% CHAPS.

Protein concentration was determined with Bio-Rad protein assay. For DIGE minimal labelling, 25 *μ*g of protein sample were mixed with 100 pmol CyDye (GE Healthcare) and incubated on ice in the dark for 30 min. The experimental design is reported in Table 1.

**Table 1:** 2D-DIGE experimental design

**Gel number**	**Cy2**	**Cy3**	**Cy5**
1	Pooled standard	Sample 1	Sample 2
2	Pooled standard	Sample 3	Sample 4
3	Pooled standard	Sample 5	-

For DIGE minimal labelling, 25 *μ*g of protein sample were mixed with 100 pmol CyDye (GE Healthcare). Every gel included 2 different samples, labelled with a different CyDye, and a Cy2-labelled internal standard, which is a pool of all the samples within the experiment.

Protein samples were then mixed with rehydration buffer (7 M urea, 2 M thiourea, 4% CHAPS, DTT 40 mM), 0.05% IPG buffer (Bio-Rad) and a trace amount of Bromophenol blue. Eleven cm immobilized pH gradient strips [pH 3–10 NL] (Bio-Rad, Milan, IT) were subjected to passive rehydration overnight at room temperature and then run on a Protean IEF Cell (Bio-Rad) as previously described [13].

Subsequent to the first dimension, and before loading onto SDS-PAGE gels, IPG strips were equilibrated in 7 M urea, 2 M thiourea, 2% SDS, 30% glycerol, 50 mM Tris-HCl pH 8.8), reduced with 65 mM DTT and alkylated with 135 mM iodoacetamide. The second dimension was run on Criterion IPG + 1 Comb 8–16% precast gels (Bio-Rad). Gels were scanned on a Typhoon TRIO scanner (GE Healthcare) at 100 μm resolution. Images were subjected to Difference In-gel Analysis (DIA) using DeCyder software version 6.5 (GE Healthcare) which normalizes and statistically analyzes protein spots.

Preparative gel has been obtained, through the above described procedure, but with a 300 μg total protein load. After 2-DE the gel was fixed in 50% ethanol and 2% ortophosphoric acid followed by an exposure to a staining solution (17%(NH_4_)_2_SO_4_, 2%ortophosphoric acid, 34%methanol). Coomassie Colloidal G-250 was added to a final concentration of 0.065%. Destaining of the gel was performed with MilliQ water until the background was completely clear. Coomassie-stained gel was scanned with a GS-800 densitometer (Bio-Rad) at 63 μm resolution.

### Identification of Protein Spots using MALDI-TOF mass spectrometry

Protein spots were excised and destained with 25 mM ammonium bicarbonate in 50% acetonitrile till the gels were changed opaque and colorless. After overnight in-gel trypsin digestion, peptide mixtures were extracted with 1% triflouroacetic acid (TFA), subjected to ZipTip clean-up (Millipore SPA, Milan, Italy) according to the manufacturer's instructions and directly eluted with a α-Cyano-4-hydroxycinnamic acid matrix (10 g/l α-Cyano-4-hydroxycinnamic acid in 50% acetonitrile, 0.3% TFA). MALDI mass spectra were acquired with a Voyager DE Pro mass spectrometer (Applied Biosystems), in 700–4000 Da molecular weight range, in reflector and in positive ion mode, with 150 nsec delay time and an ion acceleration voltage of 20 kV. Spectra were externally calibrated using Peptide calibration Mix 4 (Proteomix) 500–3500 Da (Laser Bio Labs). Mass spectra, obtained by collecting 1000–2000 laser shots, were processed using Data Explorer version 5.1 software (Applied Biosystems). Peak lists have been obtained from the raw data following advanced baseline correction (peak width 32, flexibility 0.5, degree 0.1), noise filtering (noise filter correlation factor 0.7) and monoisotopic peak selection. Protein identification has been performed using the MASCOT search engine , Aldente  and Pro-Found  peptide mass fingerprinting (PMF) tools.

The NCBInr and Swiss Prot were used as the protein sequence databases, to produce a standardized probabilistic measure of confidence, limiting the search to human proteins, allowing for one trypsin missed cleavage and with a 150 parts per million (ppm) mass tolerance error. The fixed modification selected was cysteine carbamidomethylation, while the variable modification selected was the methionine oxidation. We included only protein identifications with a Mowse (MOlecular Weight Search) score greater than 66 (p < 0.05); the corresponding identification p-values are reported in Additional file 1.

The protein list has been analyzed with the Pathway Express tool , or searched against the Entrez Gene database, to find all associated pathways. Proteins were then grouped and listed in relationship to pathways identified.

### Average expression of each identified protein

The aim of the majority of 2D gel electrophoresis experiments is to compare two or more different samples to find changes in protein expression that reflect differences between the samples being investigated. In this study we first determined by DeCyder software analysis, normalized volumes for each protein and for each gel tested. These values have been exported to an Excel worksheet with the XML toolbox tool of DeCyder software (GE-Healthcare). Average volume, standard deviation and coefficient of variation have then been calculated for each protein spot.

Data represent the variation of a single protein expression in duodenal biopsy from each sample.

## Results and Discussion

Our sample comprised five selected individuals, from 20 adult subjects, presented at first visit at the gastroenterology unit for a suspected celiac disease. Histological and HLA analyses do not confirmed celiac disease. Accordingly, duodenal biopsies demonstrated the presence of normal lymphoid cellularity in the lamina propria, no epithelial lymphocytosis, and normal architecture of the villi and crypts.

These samples have then been used to create a human duodenum reference 2D-map that can provide an initial framework to facilitate the comparison of diseased and healthy tissues, or their response to various metabolic, biochemical, or pharmacological stimuli.

The samples were run according to the experimental design illustrated in Table 1, and then separately analyzed.

**Figure 1:**
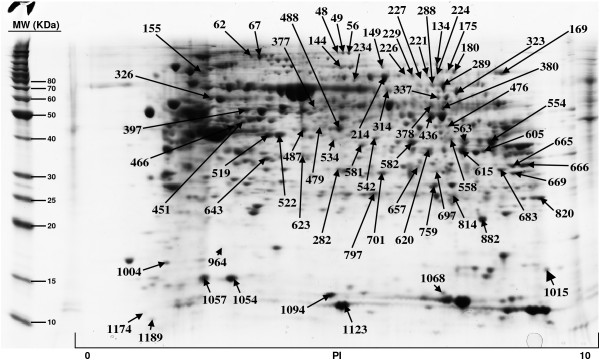
**Proteins of human duodenum tissue identified by MALDI-TOF MS from the 2-D gel**.

**Figure 2:**
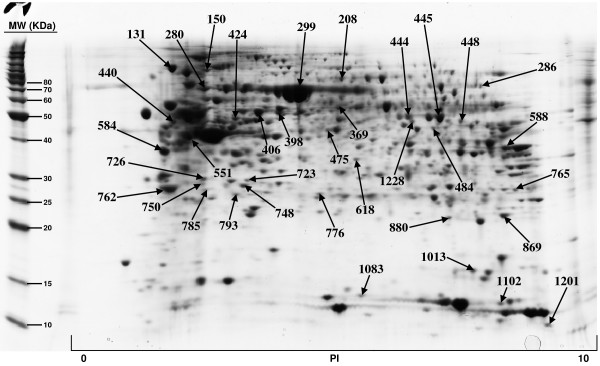
**Proteins of human duodenum tissue identified by MALDI-TOF MS from the 2-D gel**.

**Figure 3:**
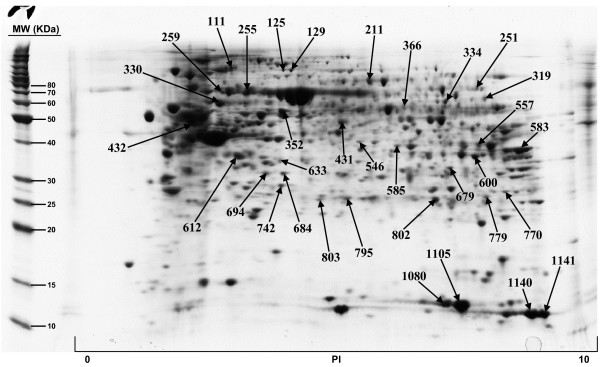
**Proteins of human duodenum tissue identified by MALDI-TOF MS from the 2-D gel**.

**Figure 4:**
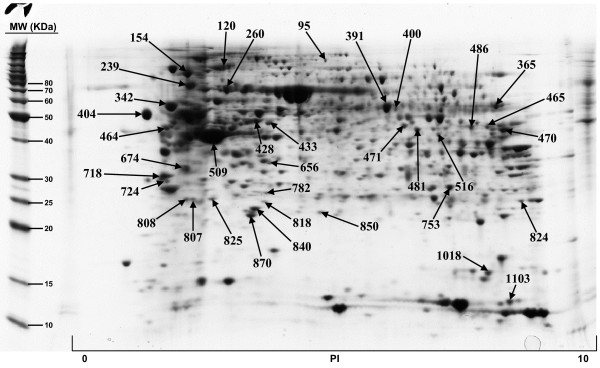
**Proteins of human duodenum tissue identified by MALDI-TOF MS from the 2-D gel**.

The included protein spots obtained from all five 2D gels, ranged from 822 to 1130. All gels were matched to the duodenum master 2D map and the normal volume of each protein spot, and for each sample, was determined by DeCyder software. The Coomassie-stained preparative gel image was then matched with analytical DIGE gel images and the visible spots, typically containing enough protein for mass spectrometry analysis, were selected for identification. A total of 179 protein spots, representing 145 unique proteins, have been so far positively identified and are illustrated in Figures 1, 2, 3, 4; quantitative data are reported on Additional file 2. We failed in identifying 39 spots. As regards faint spots the low protein amount resulted in low sequence coverage that was not sufficient for certain identifications. In the remaining 11 spots we failed in obtaining certain identifications due to the intrinsic limits of PMF: the possibility to identify only proteins for which sequences are already known and the inability to detect post-translational modifications and protein fragments.

For what regards the 179 identified proteins, we used three search engines for PMF: MASCOT, Aldente and Pro-Found. The criteria used to accept identifications were the extent of sequence coverage, the number of matching peptides and a significant probabilistic score, with at least 2 different search engines and/or both the Swiss-Prot and NCBInr databases. In the few questionable results (sequence coverage <20% and identification p-values between 0.001 and 0.05), since we could not make MS/MS analysis, we applied additional criteria to accept protein identification: (1) the search engine assignment of peptide ions must explain most of the non-background signal (2) the p*I *and molecular weight of the identified protein must be consistent with the location in the 2D gel (3) the mass error of peptide fragments must follow an internally consistent pattern as for proteins with highly significant identifications (see Additional file 3). The detailed data on all identified proteins, clustered by pathway analysis, are summarized in Additional file 1 while normal volumes, for all the identified proteins, as well as their intra-subject variations, are reported in Additional file 2.

Gel to gel variation due to differences in electrophoretic conditions, different first dimension strips and second dimension gels, gel distortions and user to user variations represent important limits for the use of a generalized protein map. The normalization of the spot amount is also questionable as the individual expression profile could be very changeable and, in addition, extractive and technical procedures can introduce further quantitative variability. Despite these limits, a dataset of small intestine proteins could be of considerable value for future studies aimed at understanding the molecular bases of small intestine biology and pathophysiology. Moreover, the use of 2D-DIGE technology with the introduction of an internal standard in every gel, greatly decreases the system variation and hinders the need of technical replicates. The internal standard, which is a pool of all the samples within the experiment, and therefore contains every protein from every sample, better increases the certainty of data; it is used to match the protein patterns across gels thereby excluding the problem of inter-gel variation, a common problem with standard 2-D assays. To estimate the reproducibility of our 2D gel images, we inserted the data of the gels of the five patients into one spreadsheet and calculated the mean, the standard deviation and the coefficient of variation for each spot (See Additional file 2). Out of the total 179 protein spots identified, 157 (88%) showed a coefficient of variation <50%, while only 7 protein spots (4%) showed a coefficient of variation >70%. Thus, it can be concluded that most of the proteomic data in this report represent stable and reproducible data that are likely to be useful as reference for further studies, and that the variation observed is mainly due to an individual variability

Moreover, to take a qualitative overview of our proteomic analysis, we listed the protein spots on the basis of their involvement in shared biological pathways (See Additional file 1). Proteins have been clustered as being involved in metabolic and energetic pathways, in immune response, in the modulation of cell proliferation and apoptosis, in protein's families with structural function and in detoxification reactions.

Among these proteins some are to be mentioned for their possible role in small intestine pathologies.

To a greater extent, the identified proteins are involved in carbohydrate, lipid and protein metabolism. The knowledge regarding the control of metabolisms in the small intestine should be of interest for clinicians involved in treatment for diabetic or septic patients, or in nutrition research in humans. They could also be important for the knowledge of inherited genetic deficiencies, such as glycogen storage disease type 1 (Von Gierke disease) and the Fanconi-Bickel and glucose-galactose malabsorption syndromes [14].

Twenty-one proteins are related to a structural function. The presence of a normal mucosal architecture is of fundamental importance for small intestine physiology. A loss of villous height and the consequent atrophic mucosa are, for example, pathognomonic of coeliac disease, Crohn's disease, autoimmune enteropathy, lymphoma and adenocarcinoma [15].

We found 10 proteins related to the peroxisome proliferator-activated receptor (PPAR) signaling pathway (See Additional file 1) [16]. The PPARα, -γ, and -β/δ are ligand activated nuclear receptors with a wide range of effects on metabolism, cellular proliferation, differentiation, and the immune response. Of considerable interest, ligands for PPARγ and PPARα have a therapeutic activity in several rodent models of inflammatory and autoimmune disease, suggesting that they might have similar activity in human disease as well [17]. It has also been shown that the PPARγ can inhibit the production of several T-cell cytokines, including the classical TH1-cell cytokine IFN-γ [18], known to be involved in the autoimmune CD pathogenesis [19]. Other proteins are related to immune response and innate immunity, as immunoglobulin M chains, that were previously found to be increased in CD [6,20], and Charcot-Leyden crystal protein (CLC) that is a lysophospholipase expressed in eosinophils, basophils and in T lymphocytes [6,21].

We identified several proteins involved in detoxification reactions, as the glutathione S-transferase (GST) proteins. The duodenum and jejunum are the main sites of absorption of xenobiotics and nutrients. Natural nutritional inducers (for example, flavonoids, indols, allyl sulphides) enhance the activity of the intestinal GSH system to build up a protective barrier against noxious agents such as carcinogens and electrophilic drugs. Moreover, important drugs such as insulin, heparin, and cortisol derivatives, as well as vegetable diets, enhance GSTP and GSTA levels. GSTA and GSTP expression levels can then represent a marker to monitor the response of the differentiated mature function of the mucosal enterocytes to specific exogenous factors [22].

Five proteins are involved in apoptosis/survival pathways: phosphatidylethanolamine-binding protein 1 (PEBP1, spot 882), also known as Raf kinase inhibitory protein (RKIP), MGC29506 (spot 964), Ras-related nuclear protein (Ran) (spot 814), 14-3-3 protein zeta/delta (spot 762) and galectin-3 (spot 765).

Among them, the PEPB1 and RAN proteins were particularly interesting for their involvement in cancer development.

PEBP1, an endogenous inhibitor of the Raf-MAPK kinase (MEK)-MAP kinase pathway, has emerged as a significant metastasis suppressor in a variety of human cancers, including colorectal cancer, and was recently shown to regulate the spindle checkpoint in cultured cells [23].

RAN is a small GTP binding protein belonging to the RAS superfamily that is essential for the translocation of RNA and proteins through the nuclear pore complex. The RAN protein is also involved in the control of DNA synthesis and cell cycle progression and also in the signal transduction pathway determining NF-kB-inducing kinase (NIK)-mediated NF-kB activation [24]. Recently RAN was demonstrated to be broadly overexpressed in cancer [25] as well as in damaged non neoplastic tissue as in CD patients with histological Marsh-III grade [26].

Other proteins, identifiable by 2DE, could also be important for the study of the small intestinal tumors, as the Galectin-3 and the HMGCS2 protein. Galectin-3 protein is known to enhance cell resistance to a variety of apoptotic stimuli [27] and, in colon cancer, increased levels of galectin-3 correlate with neoplastic progression [28]. HMGCS2 protein expression is down-regulated preferentially in moderately and poorly differentiated carcinomas. In addition, it is also down-regulated in 80% of small intestine Myc-independent tumors [29].

## Conclusion

The small intestine is an organ with a wide variety of functions, including not only nutrient absorption but also a protective response against antigenic and toxic compounds (or organisms) present in food. Proteomics can offer an unbiased approach to investigate global changes in protein expression. The analysis of biopsy specimens by proteomic technology might be useful for analysing pre-operative samples for diagnosis or for determining pathology sensitivity to therapy.

Our data, obtained by a 2-DIGE approach combined with MALDI-TOF ms protein identification and the use of software for protein functional research, illustrate the proteome of the normal duodenal mucosa. This dataset may be helpful in future proteomic studies, to a deeper knowledge of the mechanisms of intestinal disease pathogenesis, and may also help in finding new disease-specific biomarkers. Moreover, even if five determinations have low statistical significance, in a large series of the reported protein (88%), the protein expression values proved to be reproducible and stable enough to be used as reference for further studies. Finally, the 2D-gel image could be included into a Web database with active maps (e.g., SWISS-2DPAGE); this will consent to identify the protein, if this is reported in the database, by simply clicking a spot in the active gel image.

## Abbreviations

2-DE: two dimensional gel electrophoresis; MALDI-TOF MS: matrix assisted laser desorption ionization mass spectrometry; CD: celiac disease; HLA: human leukocyte antigen system; CHAPS: 3-[(3-cholamidopropyl)-dimethylammonio]-1-propanesulfonate hydrate; DIGE: 2-D Fluorescence Difference Gel Electrophoresis.

## Competing interests

The authors declare that they have no competing interests.

## Authors' contributions

MPS carried out the proteomic studies, the data analysis, participated in designing the study and helped to draft the manuscript; RC supplied the biopsy samples and participated in its design and coordination; MDM participated in experiments; AP participated in data analysis and helped to draft the manuscript; GT participated in coordinating and critically revised the manuscript; VC provided his expertise for the immunohistochemical and histological analysis of biopsy samples; VD conceived the investigation, supervised experiments and data analysis, and drafted the manuscript. All authors read and approved the final manuscript.

## Supplementary Material

Additional file 1 **Protein identification results and protein clustering on the basis of their involvement in shared biological pathways. **Proteins of human duodenum tissue identified by MALDI-TOF MS from the 2-D gel shown in Figure 1, 2, 3, 4. For every identified protein we reported the Swiss Prot database accession number, protein's molecular weight (MW) and isoelectric point (pI) and the identification results, comprising the number of matching peptides, the extent of sequence coverage and the identification p-value provided by Mascot search engine. We also reported protein spot numbers referring to Figures 1, 2, 3, 4. The list of identified proteins has been analyzed with the Pathway Express tool , or searched against the Entrez Gene database, to find all associated pathways. Proteins have then been grouped on the bases of their involvement in shared biological pathways. Identified proteins resulted involved in metabolic and energetic pathways, in immune response, in the modulation of cell proliferation and apoptosis, in protein's families with structural function and in detoxification reactions. Click here for file

Additional file 2 **Duodenal mucosa proteins expression levels.** To determine the average expression levels of each identified protein, we extracted, with the XML toolbox of DeCyder software, normalized volumes, for each protein, and for all the five gels tested. Average volume, standard deviation and the coefficient of variation have then been calculated for each protein spot. Click here for file

Additional file 3 **Mass spectra raw data and database search results. **For spots identifications with a sequence coverage <20% and identification p-values between 0.001 and 0.05, we reported mass spectra raw data and database search results. Mass spectra raw data include centroid mass, peak height, relative peak intensity and peak area. The database search results consist of matching peptides sequences, observed masses and of the mass errors between expected and experimentally observed masses. Click here for file
