# Supplementary material for: Two-dimensional gel proteome reference map of human small intestine
Source: Proteome Sci. 2009 Mar 19;7:10. doi: 10.1186/1477-5956-7-10 (PMC2667413; doi:10.1186/1477-5956-7-10)
Supplement: Additional file 1 — Protein identification results and protein clustering on the basis of their involvement in shared biological pathways. Proteins of human duodenum tissue identified by MALDI-TOF MS from the 2-D gel shown in Figure 1, 2, 3, 4. For every identified protein we reported the Swiss Prot database accession number, protein's molecular weight (MW) and isoelectric point (pI) and the identification results, comprising the number of matching peptides, the extent of sequence coverage and the identification p-value provided by Mascot search engine. We also reported protein spot numbers referring to Figures 1, 2, 3, 4. The list of identified proteins has been analyzed with the Pathway Express tool , or searched against the Entrez Gene database, to find all associated pathways. Proteins have then been grouped on the bases of their involvement in shared biological pathways. Identified proteins resulted involved in metabolic and energetic pathways, in immune response, in the modulation of cell proliferation and apoptosis, in protein's families with structural function and in detoxification reactions. [file 1477-5956-7-10-S1.doc]

Additional file 1. Protein identification results.

| **Protein Name** | **Spot** | **Database**  **Number** | **MW**  **(kDa)** | **pI** | **Matching** | **Coverage**  **(%)** | **Identification**  **p-value** |
| --- | --- | --- | --- | --- | --- | --- | --- |
| **Structural Function** | | | | | | | |
| Vinculin | 95 | P18206 | 124 | 5 | 18 | 14 | 2,70E-14 |
| Villin 1 | 144 | P09327 | 93 | 5,9 | 30 | 29 | 3,20E-15 |
| Ezrin | 208 | P15311 | 69 | 5,9 | 27 | 40 | 2,70E-17 |
| Lamin-A/C | 221 | P02545 | 74 | 6,6 | 28 | 42 | 1,30E-12 |
|  | 226 | P02545 | 64 | 6,7 | 15 | 28 | 9,40E-04 |
|  | 229 | P02545 | 74 | 6,6 | 22 | 36 | 4,10E-08 |
| Moesin | 234 | P26038 | 67 | 5,9 | 13 | 19 | 4,80E-04 |
| Lamin-B1 | 280 | P20700 | 67 | 5,1 | 29 | 36 | 4,30E-14 |
| WD repeat domain 1 | 314 | O75083 | 67 | 6,2 | 17 | 33 | 6,40E-08 |
| Keratin, type II cytoskeletal 8 | 397 | P05787 | 62 | 5,8 | 13 | 21 | 1,10E-05 |
|  | 406 | P05787 | 53 | 5,5 | 21 | 40 | 4,30E-15 |
| Keratin, type I cytoskeletal 20 | 424 | P35900 | 48 | 5,5 | 24 | 41 | 1,10E-14 |
|  | 428 | P35900 | 48 | 5,5 | 24 | 50 | 1,70E-16 |
| Actin-related protein 3 | 433 | P61158 | 48 | 5,6 | 14 | 27 | 1,40E-06 |
| Vimentin | 440 | P08670 | 54 | 5,1 | 25 | 63 | 2,20E-12 |
|  | 464 | P08670 | 54 | 5,1 | 23 | 49 | 1,10E-17 |
| Keratin 18 | 466 | P05783 | 47 | 5,3 | 28 | 46 | 2,70E-19 |
| Actin beta | 509 | P60709 | 39 | 5,2 | 15 | 46 | 6,90E-11 |
|  | 522 | P60709 | 40 | 5,5 | 17 | 43 | 4,10E-13 |
| Keratin, type I cytoskeletal 19 | 551 | P08727 | 44 | 5 | 32 | 61 | 1,40E-29 |
| Beta tropomyosin | 584 | P07951 | 30 | 4,7 | 14 | 36 | 1,70E-04 |
| F-actin-capping protein subunit beta | 694 | P47756 | 31 | 5,7 | 10 | 31 | 3,10E-04 |
| Actin-related protein 2/3 complex subunit 2 | 697 | O15144 | 34 | 6,8 | 16 | 34 | 6,30E-08 |
| Tropomyosin 3 | 718 | P06753 | 29 | 4,7 | 16 | 46 | 8,60E-08 |
| Tropomyosin 1 (alpha) | 724 | P09493 | 28 | 4,7 | 16 | 47 | 4,30E-09 |
| Actin gamma 1 | 748 | P63261 | 26 | 5,6 | 10 | 38 | 6,90E-08 |
| Actin beta | 785 | P60709 | 40 | 5,5 | 11 | 34 | 6,90E-06 |
|  | 793 | P60709 | 40 | 5,5 | 13 | 31 | 1,70E-09 |
| Profilin-1 | 1103 | P07737 | 15 | 8,4 | 7 | 57 | 2,20E-02 |
| **Protein Metabolism** | | | | | | | |
| Carbamoyl-phosphate synthase | 48 | P31327 | 166 | 6,3 | 9 | 5 | 3,20E-02 |
|  | 49 | P31327 | 166 | 6,3 | 16 | 9 | 5,10E-06 |
|  | 56 | P31327 | 166 | 6,3 | 32 | 22 | 2,60E-19 |
| Elongation factor 2 | 134 | P13639 | 96 | 6,4 | 16 | 16 | 1,00E-08 |
| Tryptophanyl-tRNA synthetase | 377 | P23381 | 53 | 5,8 | 16 | 28 | 1,00E-09 |
| Dihydrolipoamide dehydrogenase | 378 | P09622 | 51 | 6,5 | 10 | 14 | 3,00E-02 |
| Glutamate dehydrogenase | 380 | P00367 | 56 | 6,7 | 19 | 37 | 4,30E-13 |
| Leucine aminopeptidase 3 | 400 | P28838 | 56 | 8 | 21 | 41 | 5,50E-16 |
| Elongation factor Tu | 471 | P49411 | 50 | 7,3 | 16 | 35 | 4,30E-10 |
| Ornithine aminotransferase | 479 | P04181 | 49 | 6,6 | 10 | 22 | 1,90E-04 |
| Creatine kinase | 486 | P12532 | 43 | 7,3 | 17 | 36 | 6,90E-12 |
| Aminoacylase-1 | 487 | Q03154 | 46 | 5,8 | 16 | 38 | 6,40E-10 |
| Ornithine aminotransferase | 488 | P04181 | 49 | 6,6 | 16 | 33 | 1,00E-09 |
| Aspartate aminotransferase | 516 | P17174 | 46 | 6,5 | 21 | 60 | 4,30E-20 |
| Ornithine carbamoyltransferase | 563 | P00480 | 36 | 7,9 | 12 | 20 | 1,50E-04 |
| Proteasome activator subunit 1 | 742 | Q06323 | 29 | 5,8 | 13 | 39 | 4,30E-11 |
| Proteasome subunit, alpha type, 3 | 750 | P25788 | 28 | 5,2 | 14 | 48 | 2,20E-08 |
| Carbonic anhydrase II | 753 | P00918 | 29 | 6,9 | 7 | 27 | 4,70E-05 |
| Proteasome subunit alpha type-6 | 797 | P60900 | 28 | 6,3 | 9 | 32 | 5,90E-04 |
| Proteasome subunit beta type-4 | 818 | P28070 | 29 | 5,7 | 7 | 24 | 5,50E-03 |
| Glycine amidinotransferase | 1228 | P50440 | 45 | 6,6 | 16 | 27 | 5,50E-09 |
| **Sugar Metabolism** | | | | | | | |
| Sucrase | 62 | P14410 | 210 | 5,4 | 22 | 11 | 6,40E-06 |
|  | 67 | P14410 | 210 | 5,3 | 20 | 9 | 3,20E-07 |
| Neutral alpha glucosidase AB | 125 | Q14697 | 110 | 5,8 | 21 | 21 | 1,40E-07 |
|  | 129 | Q14697 | 110 | 5,8 | 21 | 19 | 2,20E-14 |
| Pyruvate kinase isozymes M1/M2 | 319 | P14618 | 60 | 8,2 | 13 | 16 | 5,50E-07 |
| Amylase, alpha 2A | 366 | P04746 | 58 | 6,6 | 11 | 20 | 2,90E-02 |
| Alpha-enolase | 444 | P06733 | 47 | 7 | 11 | 22 | 1,10E-06 |
|  | 445 | P06733 | 47 | 7 | 13 | 30 | 1,70E-08 |
| Tissue specific transplantation antigen P35B | 546 | Q13630 | 36 | 6,1 | 7 | 19 | 1,80E-02 |
| Fructose bisphosphate aldolase B | 554 | P05062 | 40 | 8,3 | 13 | 29 | 6,70E-04 |
|  | 557 | P05062 | 40 | 8,3 | 8 | 23 | 1,20E-03 |
| Aldose 1-epimerase | 581 | Q96C23 | 38 | 6,2 | 8 | 30 | 1,80E-04 |
| Fructose-1.6-bisphosphatase | 582 | P09467 | 37 | 6,5 | 16 | 33 | 3,20E-13 |
| Glyceraldehyde-3-phosphate dehydrogenase | 583 | P04406 | 36 | 8,3 | 10 | 26 | 6,40E-04 |
| Glyceraldehyde-3-phosphate dehydrogenase | 588 | P04406 | 36 | 8,3 | 13 | 30 | 4,30E-09 |
| Aflatoxin B1 aldehyde reductase member 3 | 620 | O95154 | 37 | 6,7 | 14 | 41 | 3,20E-07 |
| L-lactate dehydrogenase B chain | 633 | P07195 | 37 | 5,7 | 11 | 28 | 1,70E-05 |
| Phosphoglycerate mutase 1 | 759 | P18669 | 23 | 5,8 | 11 | 50 | 4,20E-05 |
| Triosephosphate isomerase 1 | 795 | P60174 | 23 | 6,4 | 9 | 30 | 1,20E-02 |
|  | 802 | P60174 | 27 | 6,4 | 14 | 48 | 4,30E-11 |
| **Lipid Metabolism** | | | | | | | |
| Very long-chain specific acyl-CoA dehydrogenase | 286 | P49748 | 71 | 8,9 | 19 | 27 | 3,40E-14 |
| Acetyl-Coenzyme A acyltransferase 2 | 470 | P42765 | 42 | 8,3 | 11 | 28 | 8,60E-06 |
| Phosphoglycerate kinase 1 |  | P00558 | 45 | 8,3 | 11 | 29 | 3,30E-04 |
| Short-chain specific acyl-CoA dehydrogenase | 542 | P16219 | 44 | 8,1 | 9 | 18 | 1,90E-03 |
| Aldehyde reductase | 585 | P14550 | 37 | 6,3 | 13 | 34 | 3,40E-09 |
| Aldo-keto reductase family 1 member B10 | 605 | O60218 | 36 | 7,1 | 7 | 20 | 2,50E-02 |
|  | 615 | O60218 | 36 | 7,7 | 11 | 32 | 4,70E-04 |
| Glycerol-3-phosphate dehydrogenase [NAD+] | 623 | P21695 | 38 | 5,8 | 11 | 22 | 8,00E-07 |
| Hydroxyacyl-coenzyme A dehydrogenase | 665 | Q16836 | 33 | 8,4 | 14 | 30 | 3,20E-07 |
| Enoyl-CoA hydratase | 776 | P30084 | 31 | 6,1 | 8 | 30 | 8,60E-05 |
| **Energy Production** | | | | | | | |
| Aconitase 1 | 149 | P21399 | 99 | 6,2 | 25 | 23 | 4,30E-16 |
| Aconitate hydratase | 175 | Q99798 | 86 | 7,6 | 9 | 13 | 2,40E-03 |
|  | 180 | Q99798 | 86 | 7,6 | 22 | 28 | 8,10E-09 |
| ATP synthase subunit alpha | 365 | P25705 | 60 | 9,2 | 13 | 20 | 1,10E-07 |
| ATP synthase subunit beta | 432 | P06576 | 48 | 4,9 | 21 | 49 | 3,40E-13 |
| Ubiquinol-cytochrome c reductase core protein II | 465 | P22695 | 48 | 8,7 | 18 | 42 | 1,10E-14 |
| Isocitrate dehydrogenase | 481 | O75874 | 47 | 6,5 | 12 | 24 | 6,90E-09 |
| NADH dehydrogenase [ubiquinone] 1  alpha subcomplex subunit 10 | 558 | O95299 | 41 | 8,7 | 6 | 11 | 1,30E-02 |
| Carbonyl reductase (NADPH) 1 | 669 | P16152 | 31 | 8,6 | 18 | 72 | 1,00E-20 |
|  | 683 | P16152 | 31 | 8,6 | 13 | 43 | 1,00E-06 |
| Electron transfer flavoprotein subunit beta | 770 | P38117 | 28 | 8,2 | 8 | 27 | 2,70E-03 |
| Cytochrome b5 | 1004 | P00167 | 11 | 5 | 7 | 79 | 2,50E-08 |
| **Immune Response** | | | | | | | |
| Endoplasmin | 131 | P14625 | 92 | 4,8 | 27 | 26 | 8,60E-16 |
| Heat shock protein HSP 90-alpha | 154 | P07900 | 85 | 5,1 | 26 | 45 | 5,40E-17 |
| Heat shock protein HSP 90-beta |  | P08238 | 83 | 5 | 25 | 33 | 1,70E-12 |
| Endoplasmin | 155 | P14625 | 93 | 4,7 | 26 | 27 | 8,10E-07 |
| Heat shock protein HSP 90-alpha |  | P07900 | 74 | 5,1 | 20 | 30 | 1,90E-04 |
| Ig mu chain C region | 224 | P01871 | 50 | 6,4 | 11 | 19 | 1,00E-04 |
|  | 227 | P01871 | 42 | 6,3 | 10 | 22 | 1,20E-02 |
| Heat shock 70kDa protein 5 | 239 | P11021 | 71 | 5,2 | 24 | 37 | 5,40E-16 |
| Heat shock cognate 71 kDa protein | 259 | P11142 | 71 | 5,3 | 18 | 27 | 1,70E-05 |
|  | 260 | P11142 | 71 | 5,3 | 24 | 35 | 1,70E-14 |
| Phosphoenolpyruvate carboxykinase | 288 | Q16822 | 71 | 7,5 | 17 | 22 | 3,20E-08 |
|  | 289 | Q16822 | 71 | 7,6 | 19 | 27 | 1,00E-08 |
| Protein disulfide isomerase A3 | 352 | P30101 | 54 | 5,6 | 27 | 46 | 1,10E-21 |
| Calreticulin | 404 | P27797 | 48 | 4,3 | 12 | 28 | 8,60E-07 |
| Hydroxymethylglutaryl-CoA synthase | 436 | P54868 | 55 | 8,7 | 12 | 16 | 1,60E-05 |
| Medium-chain specific acyl-CoA dehydrogenase | 476 | P11310 | 47 | 8,6 | 15 | 31 | 8,10E-07 |
|  | 484 | P11310 | 47 | 8,6 | 10 | 19 | 2,20E-07 |
| Apolipoprotein A-I | 825 | P02647 | 28 | 5,3 | 18 | 57 | 2,70E-14 |
| Charcot-Leyden crystal protein | 1068 | Q05315 | 16 | 6,8 | 6 | 19 | 7,10E-03 |
|  | 1083 | Q05315 | 16 | 6,8 | 8 | 31 | 1,80E-03 |
| Intestinal fatty acid binding protein | 1094 | P12104 | 15 | 6,6 | 7 | 31 | 1,90E-02 |
| Fatty acid-binding protein | 1123 | P07148 | 14 | 6,6 | 6 | 38 | 3,30E-02 |
| Apolipoprotein CIII | 1174 | P02656 | 9 | 4,7 | 3 | 46 | 2,90E-02 |
|  | 1189 | P02656 | 9 | 4,7 | 4 | 46 | 1,70E-03 |
| **Detoxification** | | | | | | | |
| Sulfotransferase 1A1 | 282 | P50225 | 34 | 6,5 | 11 | 23 | 3,80E-04 |
| Catalase | 334 | P04040 | 60 | 6,9 | 15 | 31 | 3,40E-11 |
|  | 337 | P04040 | 60 | 6,9 | 15 | 28 | 1,60E-08 |
| 3-mercaptopyruvate sulfurtransferase | 618 | P25325 | 33 | 6,1 | 8 | 16 | 5,10E-04 |
| Sulfotransferase 1A3/1A4 | 643 | P50224 | 34 | 5,7 | 16 | 56 | 1,30E-11 |
|  | 656 | P50224 | 34 | 5,7 | 9 | 18 | 3,40E-04 |
| Esterase D/formylglutathione hydrolase | 657 | P10768 | 29 | 6,3 | 8 | 25 | 2,20E-05 |
| Thiosulfate sulfurtransferase | 679 | Q16762 | 34 | 6,8 | 12 | 38 | 2,70E-07 |
| Peroxiredoxin-4. | 782 | Q13162 | 31 | 5,9 | 8 | 27 | 1,10E-03 |
| Glutathione S-transferase A1 | 820 | P08263 | 26 | 8,9 | 7 | 21 | 1,20E-02 |
|  | 824 | P08263 | 26 | 8,9 | 8 | 26 | 1,10E-03 |
| Glutathione S-transferase pi 1 | 840 | P09211 | 23 | 5,4 | 6 | 32 | 6,90E-03 |
| Peroxiredoxin 3 | 850 | P30048 | 25 | 5,7 | 6 | 23 | 1,40E-03 |
| Peroxiredoxin-1 | 869 | Q06830 | 22 | 8,3 | 11 | 51 | 3,40E-13 |
| Peroxiredoxin 2 | 870 | P32119 | 22 | 5,7 | 11 | 48 | 8,60E-09 |
| Superoxide dismutase 2 | 880 | P04179 | 20 | 7,8 | 7 | 35 | 1,10E-04 |
| **Cell Proliferation and Apoptosis** | | | | | | | |
| 14-3-3 protein zeta/delta | 762 | P63104 | 30 | 4,7 | 14 | 36 | 8,60E-06 |
| GTP-binding nuclear protein Ran | 814 | P62826 | 25 | 7,1 | 11 | 42 | 1,30E-08 |
| Phosphatidylethanolamine-binding protein 1 | 882 | P30086 | 21 | 7,4 | 12 | 56 | 1,30E-07 |
| Hypotetical protein MGC29506 | 964 | Q8WU39 | 21 | 5,4 | 8 | 28 | 3,80E-02 |
| Galectin-3 | 765 | P17931 | 26 | 8,6 | 8 | 24 | 4,30E-05 |
| **Other Proteins** | | | | | | | |
| Major vault protein | 111 | Q14764 | 99 | 5,3 | 18 | 17 | 2,70E-05 |
| Alpha-actinin-4. | 120 | O43707 | 104 | 5,2 | 24 | 25 | 1,70E-08 |
| Major vault protein |  | Q14764 | 99 | 5,3 | 18 | 20 | 1,70E-05 |
| Valosin-containing protein | 150 | P55072 | 90 | 5,1 | 15 | 14 | 2,30E-03 |
| Calcium-activated chloride channel regulator 1 | 169 | A8K7I4 | 101 | 5,9 | 17 | 14 | 1,60E-08 |
| Transferrin | 211 | P02787 | 79 | 7 | 16 | 18 | 4,30E-07 |
|  | 214 | P02787 | 79 | 6,9 | 16 | 16 | 2,50E-04 |
| Transketolase | 251 | P29401 | 68 | 7,6 | 14 | 24 | 1,40E-06 |
| Heat shock 70kDa protein 9 | 255 | P38646 | 74 | 5,9 | 22 | 33 | 2,20E-11 |
| Serum albumin | 299 | P02768 | 68 | 5,7 | 21 | 33 | 3,40E-11 |
| Dihydroxyacetone kinase | 323 | Q3LXA3 | 59 | 7,6 | 15 | 30 | 3,20E-09 |
| 60 kDa heat shock protein | 326 | P10809 | 61 | 5,7 | 13 | 24 | 6,90E-03 |
|  | 330 | P10809 | 61 | 5,8 | 14 | 29 | 3,40E-07 |
| Protein disulfide-isomerase | 342 | P07237 | 57 | 4,8 | 29 | 50 | 3,40E-24 |
| T-complex protein 1 subunit beta | 369 | P78371 | 58 | 6 | 16 | 30 | 3,70E-03 |
| Retinal dehydrogenase 1 | 391 | P00352 | 55 | 6,3 | 12 | 26 | 7,50E-05 |
| Peptidase D | 398 | P12955 | 55 | 5,6 | 14 | 22 | 7,50E-04 |
| Rab GDP dissociation inhibitor beta | 431 | P50395 | 51 | 6,1 | 19 | 31 | 1,70E-06 |
| Fumarate hydratase | 448 | P07954 | 55 | 8,8 | 16 | 30 | 5,50E-13 |
| Guanine deaminase | 451 | Q9Y2T3 | 51 | 5,4 | 12 | 28 | 9,10E-04 |
| S-adenosylhomocysteine hydrolase | 475 | P23526 | 48 | 5,9 | 15 | 30 | 1,10E-11 |
| Adenosine deaminase | 519 | P00813 | 41 | 5,6 | 13 | 29 | 2,60E-07 |
| DnaJ homolog subfamily B member 11 | 534 | Q9UBS4 | 41 | 5,8 | 10 | 28 | 7,00E-05 |
| Annexin A2 | 600 | P07355 | 39 | 7,6 | 18 | 41 | 2,70E-15 |
| Serum albumin | 612 | P02768 | 68 | 5,6 | 12 | 14 | 8,60E-04 |
| Voltage-dependent anion-selective channel protein 1 | 666 | P21796 | 31 | 8,6 | 8 | 31 | 1,10E-04 |
| Annexin A5 | 674 | P08758 | 36 | 4,9 | 20 | 59 | 8,60E-15 |
| Annexin A4 | 684 | P09525 | 34 | 5,6 | 10 | 26 | 3,40E-07 |
| Purine nucleoside phosphorylase | 701 | P00491 | 32 | 6,5 | 13 | 35 | 2,00E-06 |
| Prohibitin | 723 | P35232 | 30 | 5,5 | 10 | 33 | 6,30E-05 |
| Chloride intracellular channel 1 | 726 | O00299 | 24 | 5,1 | 8 | 45 | 2,30E-05 |
| 3-hydroxybutyrate dehydrogenase type 2 | 779 | Q9BUT1 | 27 | 7,6 | 9 | 28 | 1,60E-04 |
| Heat shock protein beta-1 | 803 | P04792 | 23 | 6 | 11 | 53 | 2,20E-08 |
| Rho GDP dissociation inhibitor (GDI) alpha | 807 | P52565 | 23 | 5 | 12 | 46 | 1,40E-08 |
|  | 808 | P52565 | 23 | 5 | 11 | 40 | 8,60E-08 |
| Peptidylprolyl isomerase A | 1013 | P62937 | 18 | 7,7 | 12 | 43 | 1,10E-04 |
| Anterior gradient protein 2 homolog | 1015 | O95994 | 20 | 9 | 6 | 32 | 7,80E-03 |
| Peptidylprolyl isomerase A | 1018 | P62937 | 18 | 7,7 | 9 | 40 | 2,70E-06 |
| Retinol binding protein II | 1054 | P50120 | 16 | 5,3 | 7 | 42 | 2,60E-02 |
|  | 1057 | P50120 | 16 | 5,3 | 5 | 35 | 1,40E-02 |
| Hemoglobin subunit beta | 1080 | P68871 | 14 | 6,5 | 8 | 71 | 2,20E-06 |
| Hemoglobin, delta | 1102 | P02042 | 16 | 7,8 | 9 | 66 | 8,60E-07 |
| Hemoglobin subunit beta | 1105 | P68871 | 14 | 6,5 | 7 | 71 | 2,20E-05 |
| Hemoglobin subunit alpha | 1140 | P69905 | 11 | 7,1 | 6 | 55 | 3,10E-03 |
|  | 1141 | P69905 | 11 | 7,1 | 5 | 55 | 1,50E-02 |
| Heat shock 10kDa protein 1 | 1201 | P61604 | 9 | 8,9 | 5 | 40 | 3,60E-03 |
