# Supplementary material for: Two-dimensional gel proteome reference map of human small intestine
Source: Proteome Sci. 2009 Mar 19;7:10. doi: 10.1186/1477-5956-7-10 (PMC2667413; doi:10.1186/1477-5956-7-10)
Supplement: Additional file 2 — Duodenal mucosa proteins expression levels. To determine the average expression levels of each identified protein, we extracted, with the XML toolbox of DeCyder software, normalized volumes, for each protein, and for all the five gels tested. Average volume, standard deviation and the coefficient of variation have then been calculated for each protein spot. [file 1477-5956-7-10-S2.doc]

Additional file 2. Duodenal mucosa proteins expression levels.

| **Protein name** | **Spot**  **number** | **Accession**  **number** | **Gel 1** | **Gel 2** | **Gel 3** | **Gel 4** | **Gel 5** | **Mean** | **SD** | **CV%** |
| --- | --- | --- | --- | --- | --- | --- | --- | --- | --- | --- |
| Carbamoyl-phosphate synthase | 48 | P31327 | 0,75 | 1,11 | 0,55 | 0,32 | 1,08 | 0,76 | 0,34 | 45 |
| Carbamoyl-phosphate synthase | 49 | P31327 | 1,86 | 2,60 | 1,98 | 0,55 | 2,24 | 1,84 | 0,78 | 42 |
| Carbamoyl-phosphate synthase | 56 | P31327 | 3,48 | 4,46 | 4,49 | 1,39 | 4,11 | 3,59 | 1,29 | 36 |
| Sucrase | 62 | P14410 | 2,28 | 2,01 | 2,37 | 1,87 | 1,10 | 1,93 | 0,50 | 26 |
| Sucrase | 67 | P14410 | 4,65 | 3,11 | 8,26 | 5,10 | 3,28 | 4,88 | 2,08 | 43 |
| Vinculin | 95 | P18206 | 1,49 | 1,11 | 1,79 | 0,47 | 1,36 | 1,24 | 0,50 | 40 |
| Major vault protein | 111 | Q14764 | 4,02 | 4,81 | 9,02 | 3,08 | 9,67 | 6,12 | 3,01 | 49 |
| Alpha-actinin-4/Major vault protein | 120 | O43707/Q14764 | 6,62 | 6,61 | 8,25 | 3,85 | 8,99 | 6,86 | 1,98 | 29 |
| Neutral alpha glucosidase AB | 125 | Q14697 | 0,99 | 0,80 | 0,72 | 0,82 | 0,58 | 0,78 | 0,15 | 19 |
| Neutral alpha glucosidase AB | 129 | Q14697 | 1,99 | 1,94 | 2,60 | 1,78 | 1,69 | 2,00 | 0,36 | 18 |
| Endoplasmin | 131 | P14625 | 14,48 | 21,12 | 38,15 | 14,08 | 20,46 | 21,66 | 9,78 | 45 |
| Elongation factor 2 | 134 | P13639 | 2,30 | 1,47 | 1,58 | 0,71 | 1,48 | 1,51 | 0,56 | 37 |
| Villin-1 | 144 | P09327 | 1,47 | 1,91 | 1,75 | 1,57 | 1,19 | 1,58 | 0,28 | 17 |
| Iron-responsive element-binding protein 1 | 149 | P21399 | 2,58 | 2,99 | 4,69 | 1,66 | 3,57 | 3,10 | 1,13 | 36 |
| Valosin-containing protein | 150 | P55072 | 4,63 | 4,46 | 1,94 | 1,83 | 1,53 | 2,88 | 1,53 | 53 |
| Heat shock protein HSP 90-alpha/beta | 154 | P07900/P08238 | 10,09 | 13,93 | 40,41 | 2,71 | 10,26 | 15,48 | 14,52 | 94 |
| Endoplasmin/Heat shock protein 90 alpha | 155 | P14625/P07900 | 2,73 | 3,29 | 0,96 | 2,48 | 2,39 | 2,37 | 0,86 | 36 |
| Chloride channel calcium activated family member 1 | 169 | A8K7I4 | 22,65 | 10,37 | 2,91 | 1,61 | 3,03 | 8,11 | 8,83 | 109 |
| Aconitate hydratase | 175 | Q99798 | 2,67 | 1,77 | 0,76 | 0,87 | 2,31 | 1,67 | 0,85 | 51 |
| Aconitate hydratase | 180 | Q99798 | 6,10 | 4,11 | 1,62 | 2,74 | 5,97 | 4,11 | 1,97 | 48 |
| Ezrin | 208 | P15311 | 3,25 | 5,80 | 1,84 | 1,69 | 3,55 | 3,22 | 1,66 | 51 |
| Transferrin | 211 | P02787 | 6,56 | 5,02 | 10,02 | 5,95 | 7,50 | 7,01 | 1,91 | 27 |
| Serotransferrin | 214 | P02787 | 9,80 | 7,67 | 15,27 | 9,40 | 14,25 | 11,28 | 3,30 | 29 |
| Lamin-A/C | 221 | P02545 | 1,15 | 0,84 | 1,19 | 0,69 | 1,10 | 0,99 | 0,22 | 22 |
| Ig mu chain C region | 224 | P01871 | 2,83 | 1,36 | 1,79 | 1,49 | 2,12 | 1,92 | 0,59 | 31 |
| Lamin A/C | 226 | P02545 | 1,01 | 1,33 | 2,16 | 1,24 | 0,46 | 1,24 | 0,62 | 50 |
| Ig mu chain C region | 227 | P01871 | 0,47 | 0,33 | 0,72 | 0,39 | 0,58 | 0,50 | 0,16 | 32 |
| Lamin-A/C | 229 | P02545 | 2,19 | 1,50 | 2,42 | 1,17 | 2,76 | 2,01 | 0,66 | 33 |
| Moesin | 234 | P26038 | 0,75 | 0,73 | 0,80 | 0,23 | 0,93 | 0,69 | 0,27 | 39 |
| Heat shock 70kDa protein 5 | 239 | P11021 | 17,16 | 28,11 | 38,93 | 14,11 | 38,54 | 27,37 | 11,61 | 42 |
| Transketolase | 251 | P29401 | 3,57 | 1,56 | 1,44 | 1,68 | 1,73 | 2,00 | 0,89 | 44 |
| Heat shock 70kDa protein 9 | 255 | P38646 | 13,32 | 12,04 | 25,79 | 19,02 | 12,01 | 16,43 | 5,97 | 36 |
| Heat shock cognate 71 kDa protein | 259 | P11142 | 3,40 | 5,11 | 7,06 | 9,67 | 10,15 | 7,08 | 2,90 | 41 |
| Heat shock cognate 71 kDa protein | 260 | P11142 | 13,69 | 18,36 | 20,80 | 17,57 | 11,78 | 16,44 | 3,65 | 22 |
| Lamin-B1 | 280 | P20700 | 6,41 | 6,73 | 11,39 | 4,26 | 4,13 | 6,58 | 2,94 | 45 |
| Sulfotransferase 1A1 | 282 | P50225 | 0,70 | 0,60 | 0,53 | 0,18 | 0,59 | 0,52 | 0,20 | 38 |
| Very long-chain specific acyl-CoA dehydrogenase | 286 | P25705 | 0,88 | 0,33 | 0,29 | 0,39 | 0,63 | 0,50 | 0,25 | 50 |
| Phosphoenolpyruvate carboxykinase | 288 | Q16822 | 1,98 | 2,80 | 0,86 | 1,16 | 2,17 | 1,79 | 0,79 | 44 |
| Phosphoenolpyruvate carboxykinase | 289 | Q16822 | 6,27 | 2,59 | 3,06 | 4,93 | 7,10 | 4,79 | 1,96 | 41 |
| Serum albumin | 299 | P02768 | 20,90 | 29,12 | 26,98 | 26,31 | 37,26 | 28,11 | 5,94 | 21 |
| WD repeat domain 1 | 314 | O75083 | 2,16 | 2,02 | 4,19 | 2,15 | 3,64 | 2,83 | 1,01 | 36 |
| Pyruvate kinase isozymes M1/M2 | 319 | P14618 | 4,52 | 4,81 | 2,12 | 0,91 | 5,29 | 3,53 | 1,91 | 54 |
| Dihydroxyacetone kinase | 323 | Q3LXA3 | 4,76 | 4,02 | 2,54 | 2,97 | 3,65 | 3,59 | 0,87 | 24 |
| 60 kDa heat shock protein | 326 | P10809 | 3,41 | 4,42 | 5,30 | 6,09 | 5,79 | 5,00 | 1,10 | 22 |
| 60 kDa heat shock protein | 330 | P10809 | 13,64 | 17,83 | 17,48 | 14,72 | 13,15 | 15,36 | 2,17 | 14 |
| Catalase | 334 | P04040 | 4,29 | 3,28 | 2,90 | 2,99 | 3,74 | 3,44 | 0,58 | 17 |
| Catalase | 337 | P04040 | 3,44 | 2,60 | 1,04 | 1,81 | 3,45 | 2,47 | 1,05 | 43 |
| Protein disulfide-isomerase | 342 | P07237 | 22,69 | 30,10 | 38,27 | 31,52 | 40,89 | 32,69 | 7,19 | 22 |
| Protein disulfide isomerase A3 | 352 | P30101 | 11,45 | 15,05 | 14,14 | 11,23 | 16,52 | 13,68 | 2,30 | 17 |
| ATP synthase subunit alpha | 365 | P48735 | 16,76 | 15,62 | 14,99 | 13,91 | 23,79 | 17,01 | 3,93 | 23 |
| Amylase, alpha 2A | 366 | P04746 | 1,93 | 0,91 | 0,86 | 2,51 | 2,55 | 1,75 | 0,83 | 47 |
| T-complex protein 1 subunit beta | 369 | P78371 | 3,03 | 3,99 | 3,32 | 2,08 | 2,16 | 2,92 | 0,80 | 28 |
| Tryptophanyl-tRNA synthetase | 377 | P23381 | 2,11 | 2,26 | 1,63 | 1,10 | 1,32 | 1,68 | 0,50 | 30 |
| Dihydrolipoamide dehydrogenase | 378 | P09622 | 8,34 | 7,13 | 6,03 | 4,41 | 10,51 | 7,28 | 2,31 | 32 |
| Glutamate dehydrogenase | 380 | P00367 | 6,81 | 6,60 | 4,19 | 5,10 | 6,60 | 5,86 | 1,16 | 20 |
| Retinal dehydrogenase 1 | 391 | P00352 | 20,27 | 23,42 | 21,50 | 17,35 | 17,25 | 19,96 | 2,68 | 13 |
| Keratin 8 | 397 | P05787 | 2,83 | 9,87 | 9,82 | 4,31 | 6,71 | 6,71 | 3,18 | 47 |
| Peptidase D | 398 | P12955 | 8,46 | 8,94 | 5,32 | 7,55 | 5,92 | 7,24 | 1,57 | 22 |
| Leucine aminopeptidase 3 | 400 | P28838 | 4,99 | 4,64 | 4,42 | 9,45 | 4,27 | 5,55 | 2,19 | 39 |
| Calreticulin | 404 | P27797 | 12,56 | 17,47 | 23,06 | 18,80 | 16,21 | 17,62 | 3,83 | 22 |
| Keratin, type II cytoskeletal 8 | 406 | P05787 | 8,04 | 15,56 | 20,50 | 13,10 | 17,71 | 14,98 | 4,74 | 32 |
| Keratin, type I cytoskeletal 20 | 424 | P35900 | 2,61 | 5,59 | 4,58 | 4,49 | 5,57 | 4,57 | 1,22 | 27 |
| Keratin, type I cytoskeletal 20 | 428 | P35900 | 3,33 | 4,04 | 5,03 | 6,79 | 3,78 | 4,59 | 1,38 | 30 |
| Rab GDP dissociation inhibitor beta | 431 | P50395 | 3,75 | 2,79 | 3,19 | 1,62 | 2,04 | 2,68 | 0,86 | 32 |
| ATP synthase subunit beta | 432 | P06576 | 9,95 | 19,90 | 34,71 | 30,44 | 18,47 | 22,69 | 9,90 | 44 |
| Actin-related protein 3 | 433 | P61158 | 3,03 | 3,04 | 3,39 | 3,15 | 3,18 | 3,16 | 0,15 | 5 |
| Hydroxymethylglutaryl-CoA synthase | 436 | P54868 | 8,34 | 10,90 | 7,28 | 10,01 | 12,04 | 9,72 | 1,92 | 20 |
| Vimentin | 440 | P08670 | 3,25 | 1,94 | 2,81 | 3,80 | 5,67 | 3,49 | 1,40 | 40 |
| Alpha-enolase | 444 | P06733 | 5,73 | 3,80 | 4,76 | 7,87 | 4,86 | 5,40 | 1,54 | 28 |
| Alpha enolase | 445 | P06733 | 14,62 | 17,37 | 21,20 | 21,73 | 19,76 | 18,93 | 2,94 | 16 |
| Fumarate hydratase | 448 | P07954 | 3,15 | 2,18 | 2,47 | 2,92 | 2,40 | 2,62 | 0,40 | 15 |
| Guanine deaminase | 451 | Q9Y2T3 | 3,39 | 4,51 | 3,71 | 2,18 | 2,83 | 3,33 | 0,88 | 27 |
| Vimentin | 464 | P08670 | 2,99 | 1,74 | 3,27 | 3,75 | 2,52 | 2,86 | 0,77 | 27 |
| Ubiquinol-cytochrome c reductase core protein II | 465 | P22695 | 5,45 | 4,44 | 4,66 | 4,60 | 4,49 | 4,73 | 0,41 | 9 |
| Keratin 18 | 466 | P05783 | 2,82 | 4,45 | 3,39 | 0,61 | 6,89 | 3,63 | 2,30 | 63 |
| Acetyl-Coenzyme A acyltransferase 2/Phosphoglycerate kinase 1 | 470 | P42765/P00558 | 30,83 | 17,20 | 17,17 | 23,57 | 17,68 | 21,29 | 5,98 | 28 |
| Elongation factor Tu | 471 | P49411 | 3,83 | 3,57 | 3,28 | 3,06 | 3,39 | 3,43 | 0,29 | 9 |
| S-adenosylhomocysteine hydrolase | 475 | P23526 | 1,98 | 2,36 | 1,89 | 0,78 | 1,19 | 1,64 | 0,64 | 39 |
| Medium-chain specific acyl-CoA dehydrogenase | 476 | P11310 | 1,23 | 0,70 | 0,90 | 1,22 | 1,29 | 1,07 | 0,26 | 24 |
| Ornithine aminotransferase | 479 | P04181 | 1,43 | 1,30 | 0,77 | 1,24 | 0,70 | 1,09 | 0,33 | 30 |
| Isocitrate dehydrogenase | 481 | O75874 | 7,64 | 7,31 | 8,14 | 6,67 | 9,36 | 7,82 | 1,01 | 13 |
| Medium-chain specific acyl-CoA dehydrogenase | 484 | P11310 | 3,22 | 2,19 | 1,64 | 2,37 | 3,07 | 2,50 | 0,65 | 26 |
| Creatine kinase | 486 | P12532 | 3,14 | 2,68 | 2,88 | 4,13 | 4,25 | 3,42 | 0,72 | 21 |
| Aminoacylase-1 | 487 | Q03154 | 2,82 | 3,38 | 2,79 | 3,33 | 2,15 | 2,89 | 0,50 | 17 |
| Ornithine aminotransferase; hepatic form | 488 | P04181 | 6,48 | 5,08 | 7,00 | 3,24 | 4,10 | 5,18 | 1,58 | 30 |
| Actin, beta | 509 | P60709 | 16,00 | 7,06 | 45,96 | 29,14 | 55,71 | 30,77 | 20,22 | 66 |
| Aspartate aminotransferase | 516 | P17174 | 1,69 | 1,01 | 1,18 | 0,89 | 0,75 | 1,10 | 0,36 | 33 |
| Adenosine deaminase | 519 | P00813 | 6,34 | 4,36 | 5,95 | 13,92 | 3,66 | 6,85 | 4,11 | 60 |
| Actin | 522 | P60709 | 3,04 | 2,28 | 1,82 | 18,01 | 1,20 | 5,27 | 7,15 | 136 |
| DnaJ homolog subfamily B member 11 | 534 | Q9UBS4 | 1,19 | 1,40 | 2,01 | 1,40 | 0,95 | 1,39 | 0,39 | 28 |
| Short-chain specific acyl-CoA dehydrogenase | 542 | P16219 | 2,97 | 2,62 | 3,02 | 2,00 | 3,28 | 2,78 | 0,49 | 18 |
| Tissue specific transplantation antigen P35B | 546 | Q13630 | 1,15 | 0,62 | 0,98 | 0,65 | 0,89 | 0,86 | 0,22 | 26 |
| Keratin, type I cytoskeletal 19 | 551 | P08727 | 3,92 | 1,34 | 2,99 | 2,58 | 1,93 | 2,55 | 0,99 | 39 |
| Fructose-bisphosphate aldolase B | 554 | P05062 | 9,02 | 11,83 | 10,06 | 19,15 | 14,27 | 12,86 | 4,04 | 31 |
| Fructose-bisphosphate aldolase B | 557 | P05062 | 7,39 | 4,65 | 4,59 | 6,38 | 5,52 | 5,71 | 1,19 | 21 |
| NADH dehydrogenase [ubiquinone] 1 alpha subcomplex subunit 1 | 558 | O95299 | 3,12 | 1,16 | 2,03 | 2,35 | 1,68 | 2,07 | 0,74 | 36 |
| Ornithine carbamoyltransferase | 563 | P00480 | 5,04 | 3,12 | 5,25 | 4,57 | 5,73 | 4,74 | 1,00 | 21 |
| Aldose 1-epimerase | 581 | Q96C23 | 3,44 | 3,89 | 2,05 | 1,74 | 1,74 | 2,57 | 1,02 | 39 |
| fructose-1.6-bisphosphatase | 582 | P09467 | 10,15 | 8,10 | 5,52 | 6,60 | 7,15 | 7,50 | 1,75 | 23 |
| Glyceraldehyde-3-phosphate dehydrogenase | 583 | P04406 | 19,37 | 18,57 | 20,86 | 15,86 | 20,23 | 18,98 | 1,95 | 10 |
| Beta tropomyosin | 584 | P07951 | 7,27 | 7,19 | 8,23 | 7,20 | 6,63 | 7,30 | 0,58 | 8 |
| Aldehyde reductase | 585 | P14550 | 6,57 | 4,46 | 3,11 | 3,48 | 5,63 | 4,65 | 1,45 | 31 |
| Glyceraldehyde-3-phosphate dehydrogenase | 588 | P04406 | 17,65 | 13,58 | 10,70 | 7,21 | 9,09 | 11,65 | 4,09 | 35 |
| Annexin A2 | 600 | P07355 | 9,42 | 9,57 | 8,22 | 5,53 | 7,23 | 8,00 | 1,68 | 21 |
| Aldo-keto reductase family1 B10 | 605 | O60218 | 9,62 | 2,66 | 2,43 | 2,48 | 2,08 | 3,85 | 3,23 | 84 |
| Serum albumin | 612 | P02768 | 1,76 | 0,86 | 0,47 | 10,17 | 3,37 | 3,32 | 3,98 | 120 |
| Aldo-keto reductase family 1 member B10 | 615 | O60218 | 9,33 | 20,05 | 13,86 | 14,91 | 13,90 | 14,41 | 3,82 | 27 |
| 3-mercaptopyruvate sulfurtransferase | 618 | P25325 | 1,33 | 2,39 | 2,27 | 1,07 | 1,47 | 1,70 | 0,59 | 35 |
| Aflatoxin B1 aldehyde reductase member 3 | 620 | O95154 | 4,51 | 3,37 | 5,71 | 3,34 | 6,90 | 4,77 | 1,54 | 32 |
| Glycerol-3-phosphate dehydrogenase [NAD+] | 623 | P21695 | 4,73 | 2,40 | 1,92 | 2,15 | 2,00 | 2,64 | 1,18 | 45 |
| L-lactate dehydrogenase B chain | 633 | P07195 | 2,03 | 1,33 | 1,88 | 1,79 | 1,22 | 1,65 | 0,35 | 21 |
| Monoamine-sulfating phenol sulfotransferase | 643 | P50224 | 3,08 | 2,44 | 4,57 | 2,95 | 2,69 | 3,15 | 0,83 | 26 |
| Sulfotransferase 1A3/1A4 | 656 | P50224 | 0,58 | 0,45 | 0,27 | 0,46 | 0,18 | 0,39 | 0,16 | 41 |
| Esterase D | 657 | P10768 | 2,22 | 2,49 | 2,22 | 2,88 | 3,24 | 2,61 | 0,45 | 17 |
| Hydroxyacyl-coenzyme A dehydrogenase | 665 | Q16836 | 5,55 | 2,41 | 3,38 | 3,46 | 1,51 | 3,26 | 1,51 | 46 |
| Voltage-dependent anion-selective channel protein 1 | 666 | P21796 | 21,48 | 16,39 | 7,73 | 16,87 | 7,93 | 14,08 | 6,04 | 43 |
| Carbonyl reductase (NADPH) 1 | 669 | P16152 | 8,47 | 7,96 | 4,96 | 8,60 | 6,83 | 7,37 | 1,51 | 21 |
| Annexin A5 | 674 | P08758 | 5,58 | 3,70 | 5,05 | 3,37 | 4,49 | 4,44 | 0,92 | 21 |
| Thiosulfate sulfurtransferase | 679 | Q16762 | 4,68 | 3,28 | 3,41 | 2,80 | 3,52 | 3,54 | 0,69 | 20 |
| Carbonyl Reductase | 683 | P16152 | 4,73 | 3,68 | 0,90 | 2,49 | 2,18 | 2,80 | 1,47 | 52 |
| Annexin A4 | 684 | P09525 | 4,61 | 4,76 | 4,21 | 3,99 | 2,88 | 4,09 | 0,74 | 18 |
| F-actin-capping protein subunit beta | 694 | P47756 | 1,74 | 0,74 | 2,46 | 2,01 | 2,05 | 1,80 | 0,65 | 36 |
| Actin-related protein 2/3 complex subunit 2 | 697 | O15144 | 2,53 | 2,26 | 1,68 | 2,65 | 2,09 | 2,24 | 0,38 | 17 |
| Purine nucleoside phosphorylase | 701 | P00491 | 3,43 | 1,92 | 4,90 | 3,13 | 2,02 | 3,08 | 1,22 | 40 |
| Tropomyosin 3 | 718 | P06753 | 4,33 | 4,81 | 4,43 | 4,21 | 4,57 | 4,47 | 0,23 | 5 |
| Prohibitin | 723 | P35232 | 2,00 | 0,47 | 1,13 | 0,67 | 0,77 | 1,01 | 0,60 | 60 |
| Tropomyosin 1 (alpha) | 724 | P09493 | 1,04 | 0,77 | 0,66 | 0,91 | 0,79 | 0,83 | 0,15 | 17 |
| Chloride intracellular channel 1 | 726 | O00299 | 0,47 | 2,16 | 1,41 | 1,63 | 0,93 | 1,32 | 0,65 | 49 |
| Proteasome activator subunit 1 | 742 | Q06323 | 3,47 | 2,84 | 4,45 | 4,70 | 2,40 | 3,57 | 0,99 | 28 |
| Actin gamma 1 | 748 | P63261 | 1,40 | 1,38 | 0,81 | 3,76 | 0,33 | 1,54 | 1,32 | 86 |
| Proteasome subunit, alpha type, 3 | 750 | P25788 | 0,64 | 0,84 | 0,80 | 0,86 | 0,54 | 0,74 | 0,14 | 19 |
| Carbonic anhydrase II | 753 | P00918 | 7,26 | 6,13 | 6,42 | 5,36 | 9,63 | 6,96 | 1,64 | 24 |
| Phosphoglycerate mutase 1 | 759 | P18669 | 4,51 | 4,44 | 5,61 | 5,53 | 7,70 | 5,56 | 1,32 | 24 |
| 14-3-3 protein zeta/delta | 762 | P63104 | 17,87 | 9,96 | 12,31 | 14,87 | 13,51 | 13,71 | 2,95 | 22 |
| Galectin-3 | 765 | P17931 | 2,42 | 1,76 | 2,02 | 1,67 | 1,74 | 1,92 | 0,31 | 16 |
| Electron transfer flavoprotein subunit beta | 770 | P38117 | 2,35 | 0,74 | 2,27 | 1,84 | 2,32 | 1,90 | 0,68 | 36 |
| Enoyl-CoA hydratase | 776 | P30084 | 3,85 | 3,19 | 4,55 | 3,26 | 2,40 | 3,45 | 0,80 | 23 |
| 3-hydroxybutyrate dehydrogenase type 2 | 779 | Q9BUT1 | 3,51 | 1,42 | 1,55 | 2,81 | 0,92 | 2,04 | 1,08 | 53 |
| Peroxiredoxin-4 | 782 | Q13162 | 0,82 | 0,56 | 1,27 | 0,60 | 0,86 | 0,82 | 0,28 | 34 |
| Actin beta | 785 | P60709 | 0,63 | 0,37 | 0,98 | 1,42 | 0,34 | 0,75 | 0,46 | 61 |
| Actin beta | 793 | P60709 | 1,74 | 0,88 | 1,59 | 4,03 | 0,99 | 1,85 | 1,28 | 69 |
| Triosephosphate isomerase 1 | 795 | P60174 | 2,07 | 2,04 | 3,12 | 2,31 | 1,48 | 2,20 | 0,60 | 27 |
| Proteasome subunit alpha type-6 | 797 | P60900 | 1,60 | 1,13 | 2,48 | 1,41 | 1,26 | 1,57 | 0,53 | 34 |
| Triosephosphate isomerase 1 | 802 | P60174 | 8,93 | 6,66 | 8,99 | 7,29 | 7,45 | 7,86 | 1,04 | 13 |
| Heat shock protein beta-1 | 803 | P04792 | 1,59 | 0,89 | 1,02 | 0,95 | 0,76 | 1,04 | 0,32 | 31 |
| Rho GDP dissociation inhibitor (GDI) alpha | 807 | P52565 | 2,69 | 1,71 | 2,80 | 3,02 | 4,93 | 3,03 | 1,17 | 39 |
| Rho GDP dissociation inhibitor (GDI) alpha | 808 | P52565 | 3,85 | 1,76 | 2,60 | 1,11 | 1,31 | 2,12 | 1,12 | 53 |
| GTP-binding nuclear protein Ran | 814 | P62826 | 5,02 | 4,00 | 7,78 | 5,87 | 5,87 | 5,71 | 1,39 | 24 |
| Proteasome subunit beta type-4 | 818 | P28070 | 0,31 | 0,28 | 0,65 | 0,24 | 0,40 | 0,37 | 0,16 | 44 |
| GSTA1 | 820 | P08263 | 9,76 | 3,65 | 5,57 | 4,95 | 3,76 | 5,54 | 2,49 | 45 |
| Glutathione S-transferase A1 | 824 | P08263 | 4,66 | 2,25 | 2,40 | 2,10 | 1,24 | 2,53 | 1,28 | 50 |
| Apolipoprotein A-I | 825 | P02647 | 6,44 | 1,76 | 2,22 | 4,80 | 3,36 | 3,72 | 1,92 | 52 |
| Glutathione S-transferase pi 1 | 840 | P09211 | 3,92 | 1,17 | 6,01 | 3,30 | 3,47 | 3,57 | 1,73 | 48 |
| Peroxiredoxin 3 | 850 | P30048 | 2,07 | 1,57 | 1,65 | 2,40 | 1,07 | 1,75 | 0,51 | 29 |
| Peroxiredoxin-1 | 869 | Q06830 | 4,72 | 2,70 | 5,60 | 3,87 | 3,65 | 4,11 | 1,10 | 27 |
| Peroxiredoxin 2 | 870 | P32119 | 4,78 | 2,17 | 4,96 | 2,65 | 3,81 | 3,68 | 1,25 | 34 |
| Superoxide dismutase 2 | 880 | P04179 | 2,53 | 1,79 | 2,31 | 2,97 | 2,25 | 2,37 | 0,43 | 18 |
| Phosphatidylethanolamine-binding protein 1 | 882 | P30086 | 9,04 | 6,49 | 12,02 | 14,01 | 11,91 | 10,69 | 2,94 | 28 |
| hypotetical protein MGC29506 | 964 | Q8WU39 | 0,58 | 0,29 | 0,53 | 0,81 | 0,51 | 0,54 | 0,19 | 34 |
| Cytochrome b5 | 1004 | P00167 | 1,45 | 0,76 | 1,11 | 1,66 | 0,76 | 1,15 | 0,41 | 35 |
| Peptidylprolyl isomerase A | 1013 | P62937 | 3,82 | 2,47 | 4,83 | 5,90 | 4,24 | 4,25 | 1,27 | 30 |
| Anterior gradient protein 2 homolog | 1015 | O95994 | 3,24 | 1,76 | 2,30 | 3,46 | 11,44 | 4,44 | 3,97 | 89 |
| Peptidylprolyl isomerase A | 1018 | P62937 | 7,39 | 4,32 | 8,36 | 8,14 | 6,39 | 6,92 | 1,65 | 24 |
| Retinol binding protein II | 1054 | P50120 | 9,57 | 11,15 | 14,26 | 16,73 | 16,44 | 13,63 | 3,18 | 23 |
| Retinol-binding protein II | 1057 | P50120 | 12,19 | 10,86 | 14,98 | 10,80 | 9,57 | 11,68 | 2,06 | 18 |
| Charcot-Leyden crystal protein | 1068 | Q05315 | 0,70 | 0,35 | 0,80 | 0,51 | 1,10 | 0,69 | 0,28 | 41 |
| Hemoglobin subunit beta | 1080 | P68871 | 11,45 | 4,73 | 9,83 | 11,52 | 17,96 | 11,10 | 4,73 | 43 |
| Charcot-Leyden crystal protein | 1083 | Q05315 | 1,19 | 1,26 | 4,21 | 2,10 | 1,03 | 1,96 | 1,32 | 68 |
| Fatty acid binding protein | 1094 | P12104 | 9,46 | 9,58 | 9,19 | 8,72 | 9,02 | 9,19 | 0,34 | 4 |
| Hemoglobin, delta | 1102 | P02042 | 1,71 | 1,80 | 2,66 | 3,29 | 4,01 | 2,69 | 0,98 | 36 |
| Profilin-1 | 1103 | P07737 | 2,85 | 1,65 | 2,81 | 4,74 | 4,42 | 3,29 | 1,27 | 39 |
| Hemoglobin subunit beta | 1105 | P68871 | 43,93 | 21,50 | 53,06 | 66,25 | 78,07 | 52,56 | 21,67 | 41 |
| Fatty acid-binding protein; liver | 1123 | P07148 | 32,77 | 29,53 | 52,51 | 15,82 | 26,03 | 31,33 | 13,44 | 43 |
| Hemoglobin subunit alpha | 1140 | P69905 | 31,25 | 11,08 | 22,03 | 39,32 | 40,77 | 28,89 | 12,45 | 43 |
| Hemoglobin subunit alpha | 1141 | P69905 | 28,94 | 17,34 | 39,92 | 45,45 | 46,73 | 35,68 | 12,42 | 35 |
| Apolipoprotein CIII | 1174 | P02656 | 0,90 | 0,27 | 1,29 | 1,02 | 0,62 | 0,82 | 0,39 | 48 |
| Apolipoprotein CIII | 1189 | P02656 | 1,87 | 0,67 | 1,86 | 1,30 |  | 1,43 | 0,57 | 40 |
| Heat shock 10kDa protein 1 | 1201 | P61604 | 4,65 | 4,18 | 5,90 | 5,90 | 5,58 | 5,24 | 0,79 | 15 |
| Glycine amidinotransferase | 1228 | P50440 | 12,16 | 11,12 | 9,00 | 8,58 | 9,04 | 9,98 | 1,57 | 16 |
